# Targeting GPER1 to suppress autophagy as a male-specific therapeutic strategy for iron-induced striatal injury

**DOI:** 10.1038/s41598-019-43244-0

**Published:** 2019-04-30

**Authors:** Tzu-Yun Chen, Chih-Lung Lin, Li-Fang Wang, Ke-Li Tsai, Jun-Yu Lin, Chin Hsu

**Affiliations:** 1 Department of Physiology, Faculty of Medicine, College of Medicine, Kaohsiung Medical University, Kaohsiung, Taiwan; 2 Department of Neurosurgery, Faculty of Medicine, College of Medicine, Kaohsiung Medical University, Kaohsiung, Taiwan; 3 Department of Medicinal and Applied Chemistry, College of Life Science, Kaohsiung Medical University, Kaohsiung, Taiwan; 4 Graduate Institute of Medicine, Kaohsiung Medical University, Kaohsiung, Taiwan; 5 Graduate Institute of Clinical Medicine, College of Medicine, Kaohsiung Medical University, Kaohsiung, Taiwan

**Keywords:** Neurophysiology, Endocrinology

## Abstract

The functional outcome of intracerebral hemorrhage (ICH) in young male patients are poor than in premenopausal women. After ICH, ferrous iron accumulation causes a higher level of oxidative injury associated with autophagic cell death in striatum of male mice than in females. In rodent model of ferrous citrate (FC)-infusion that simulates iron accumulation after ICH, female endogenous estradiol (E_2_) suppresses autophagy via estrogen receptor α (ERα) and contributes to less injury severity. Moreover, E_2_ implantation diminished the FC-induced autophagic cell death and injury in males, whose ERα in the striatum is less than females. Since, no sex difference of ERβ was observed in striatum, we delineated whether ERα and G-protein-coupled estrogen receptor 1 (GPER1) mediate the suppressions of FC-induced autophagy and oxidative injury by E_2_ in a sex-dimorphic manner. The results showed that the ratio of constitutive GPER1 to ERα in striatum is higher in males than in females. The GPER1 and ERα predominantly mediated suppressive effects of E_2_ on FC-induced autophagy in males and antioxidant effect of E_2_ in females, respectively. This finding opens the prospect of a male-specific therapeutic strategy targeting GPER1 for autophagy suppression in patients suffering from iron overload after hemorrhage.

## Introduction

Intracerebral hemorrhage (ICH) is a devastating disorder associated with dismal outcome. Men have poor survival than premenopausal women after ICH^1,2^, which is associated with iron accumulation and autophagy induction. Previous reports showed that males exhibited worse free radical homeostasis and a lesser defense capacity against oxidative brain damage than females did^3^. However, no effective sex-based therapy has been used in patients suffering from long-term neurodegeneration after ICH. The prevalence of ICH is expected to increase because the elderly population continues to grow, and the most serious complication of oral anticoagulation, which is given for ischemic stroke prevention, is hemorrhagic stroke^4^. Therefore, gaining insight into sex-related differences in endogenous protective mechanisms against hemorrhagic stroke will provide better strategies for optimization of sex-specific treatment.

Recently, a male-specific therapeutic strategy targeting autophagic inhibition for patients suffering from intracerebral iron overload has been suggested^5^. Although E_2_ implantation decreases FC-induced striatal injury and autophagy in both sexes^6^, how to prevent the feminizing effects of exogenous E_2_ in males is an important issue. Because the constitutive mRNA and protein levels of ERα, but not ERβ, in the striatum are higher in female than in male rats^7^, we prospect high level of endogenous E_2_ may protect against hemorrhagic stroke predominantly via ERα in females, while, in males, other ERs may mediate the neuroprotection conferred by E_2_ against FC-induced striatal injury. GPER1 has a high affinity for E_2_ and mediates both rapid signaling and transcriptional events in response to E_2_^8^. A previous report demonstrated that G1, a GPER1 agonist, had a protective effect on cognitive function via a ctivation of PI3K/Akt and downstream mTORC1^9^ that inhibited autophagy, while a GPER1 antagonist, G15, increased the level of LC3B-II and the accumulation of autophagosomes^10^. These results imply that GPER1 may mediate the suppression of FC-induced autophagy by E_2_ in males. Therefore, we hypothesize that GPER1 and ERα contribute to the neuroprotection conferred by E_2_ against FC-induced striatal injury.

## Result

### Sex differences in constitutive and inducible levels of GPER1 and ERα in the striatum

**Figure 1:**
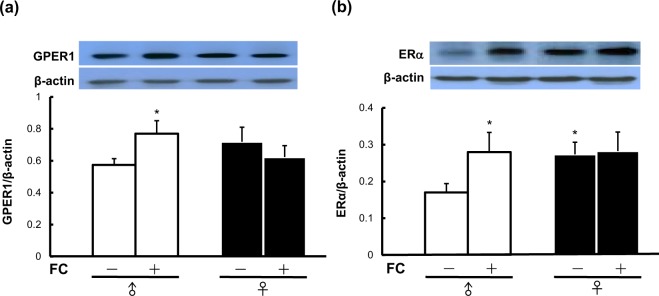
Sex differences in constitutive and inducible levels of GPER1 and ERα in the striatum. (**a**) GPER1. (**b**) ERα. Three microliters of ferrous citrate (FC) was infused into the right striatum. The constitutive and FC-induced levels of striatal GPER1 and ERα were compared between intact male and intact female mice. No sex difference in the level of constitutive GPER1, while FC-infusion increased the levels of GPER1 in males. The striatal protein level of ERα was lower in intact males than in intact females. The levels of both GPER1 and ERα were significantly increased by intrastriatal FC infusion in male mice but not in female mice. Data are expressed as the means ± s.e.m. (n = 6). *p < 0.05 compared with intact control male.

To examine the sexual dimorphism of GPER1 and ERα in the neuroprotective mechanism of E_2_ against FC-induced striatal injury, we compared the constitutive level of striatal GPER1 or ERα in intact male with that in intact female mice. Interestingly, we found that the striatal protein level of ERα was significantly lower in intact male mice than in intact females (Fig. 1a). No significant sex difference in striatal GPER1 was observed between intact mice (Fig. 1b), but the ratio of GPER1 to ERα was higher in males than in females.

To know whether FC infusion induces the expression of GPER1 or ERα in the striatum in a sex-dimorphic manner, we compared the levels of FC-induced expression of GPER1 or ERα in males with that in females. The results showed that FC infusion significantly increased the striatal levels of both GPER1 and ERα in male mice but not in female mice (Fig. 1a,b).

In order to exclude the influence of endogenous sex hormones, castrated male and female mice were used in the following studies. The protein levels of ERα and GPER1 between castrated and intact mice were compared to exclude any possible effect of castration for 2 weeks on the expression of those proteins. The results showed that there was no significant difference in the level of GPER1 or ERα between sex-matched intact mice and castrated mice (Supplement S1). To ensure that the serum level of E_2_ after implantation was within the physiological range, we measured the E_2_ concentration using an ELISA kit. As shown in Supplement S2, the serum levels of E_2_ after implantation were within the physiological range. Therefore, the following experiments were performed by using castrated male and female mice to study the effect and mechanism of exogenous E_2_ on striatal injury.

### Effects of E_2_ implantation on FC-induced behavioral deficits, striatal injury, autophagy and lipid peroxidation in castrated male and female mice

**Figure 2:**
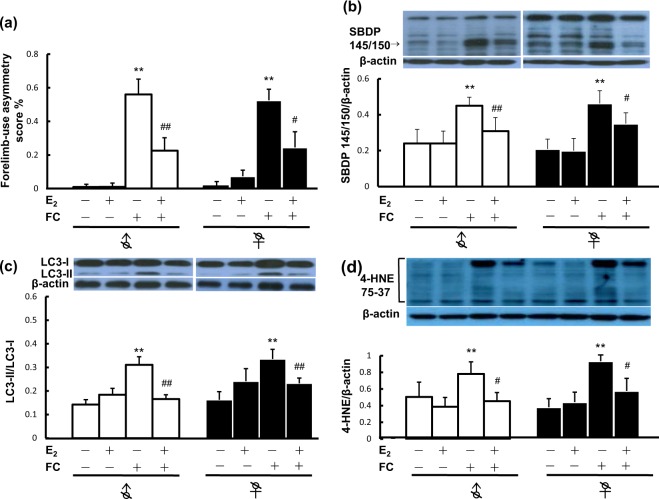
E_2_ treatment decreased FC-induced behavioral deficits, striatal injury, autophagy and lipid peroxidation in the striatum of castrated male and female mice. Orchiectomy and ovariectomy were performed 2 weeks before FC infusion. Silastic tubes containing E_2_ were implanted one day before iron infusion. Two days after FC infusion, the frequencies of right, left, and bilateral forelimb use were observed for 5 minutes. After the assessment of behavioral deficits, the striatum was sampled for Western blot analysis. White column: males; black column: females. (**a**) Behavioral deficits. The higher the forelimb-use asymmetry score, the more severe the behavioral deficit is. (**b**) Level of SBDP 145/150 (spectrin breakdown product with molecular weight of 145/150). SBDP 145/150 indicates the severity of brain injury. (**c**) LC3 lipidation. Protein levels of LC3-I and LC3-II were quantified by Western blot analysis. The ratio of LC3-II to LC3-I was estimated as a marker of autophagy. β-actin was used as a sample loading control. (**d**) Lipid peroxidation. Level of 4-HNE (4-Hydroxynonenal) was probed as a biomarker of oxidative stress, and the relative density of molecular weight between 37 and 75 was quantified after a Western blot assay. β-actin was used as a sample loading control. Data are expressed as the means ± s.e.m. (n = 6). **P < 0.01; *P < 0.05 compared with the sex-matched control group without FC infusion. ^##^*P* < 0.01; ^#^*P* < 0.05 compared with the sex-matched FC-infused group without E_2_ treatment.

To examine whether autophagy and lipid peroxidation are involved in the mechanism underlying E_2_-based protection against iron neurotoxicity in both castrated males and females, forelimb-use asymmetry scores and the levels of SBDP 145/150 were examined as indexes of behavioral deficits and the severity of striatal injury, respectively. Moreover, the ratio of LC3-II to LC3-I, which indicates LC3 lipidation and is regard as a marker for autophagy, and the level of 4-HNE, a biomarker of oxidative stress, were examined. The results showed that FC infusion increased the levels of FC-induced behavioral deficit (Fig. 2a), cleavage of α-II spectrin (Fig. 2b), LC3 lipidation (Fig. 2c) and lipid peroxidation (Fig. 2d) in both castrated male and female mice. No sex differences in FC-induced behavioral deficits (Fig. 2a) or spectrin cleavage (Fig. 2b) between castrated male and female mice were observed. Implantation of E_2_ significantly decreased the FC-induced behavioral deficits, severity of striatal injury, LC3 lipidation and lipid peroxidation in both castrated male and female mice (Fig. 2).

### Effects of E_2_, G1 (GPER1 agonist) and propyl pyrazole triol (PPT; ERα agonist) on FC-induced behavioral deficits, striatal injury, autophagy and lipid peroxidation in castrated male and female mice

**Figure 3:**
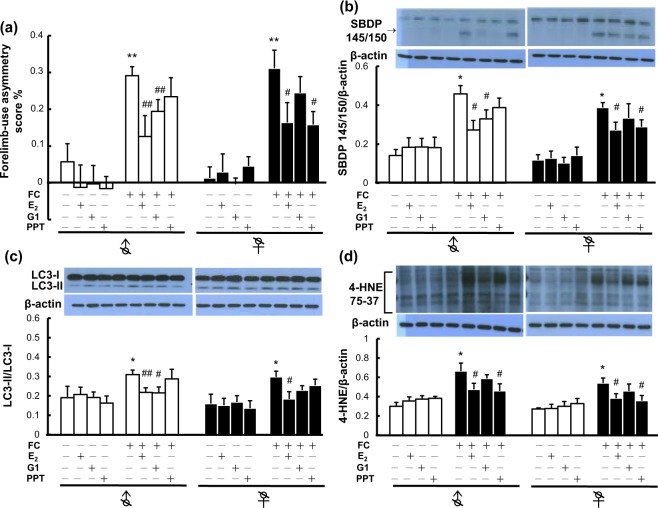
Effects of estradiol (E_2_), G1 (GPER1 agonist) and propyl pyrazole triol (PPT; ERα agonist) on FC-induced behavioral deficits (**a**), Level of SBDP 145/150 (b), LC3 lipidation (**c**), and lipid peroxidation (**d**) in castrated male and female mice. Castration was performed at 2 weeks before implantation of a Silastic tube containing E_2_, G1 or PPT. One day after implantation, FC was infused into the right striatum. Two days after FC infusion, behavior deficits were examined. Data are expressed as the means ± s.e.m. (n = 6). **P < 0.01; *P < 0.05 compared with the sex-matched control group without FC infusion. ^##^*P* < 0.01; ^#^*P* < 0.05 compared with the sex-matched FC-infused group without E_2_ treatment.

G1 and PPT were used to examine the mediating roles of GPER1 and ERα in the effect of E_2_ on FC-induced behavior deficits, injury severity, autophagy and lipid peroxidation. The results showed that E_2_ decreased the levels of FC-induced behavioral deficits, spectrin cleavage, LC3 lipidation and lipid peroxidation in the striatums of castrated male and female mice (Fig. 3a–d). G1 simulated the effects of E_2_ on the severity of FC-induced behavioral deficit (Fig. 3a), the level of FC-induced spectrin cleavage (Fig. 3b), and FC-induced LC3 lipidation (Fig. 3c) in castrated males but not in castrated females. No significant effect of G1 on FC-induced lipid peroxidation was observed in castrated males or females. On the other hand, PPT simulated the effects of E_2_ on the level of FC-induced behavior deficits in castrated female mice and the level of FC-induced lipid peroxidation in both castrated male and female mice (Fig. 3d). However, no significant effect of PPT on FC-induced behavioral deficits, spectrin cleavage, or autophagy was observed in castrated male group (Fig. 3a–c).

### Effects of silencing GPER1 or ERα on FC-induced behavioral deficits, striatal injury, autophagy, and lipid peroxidation in castrated male and female mice

**Figure 4:**
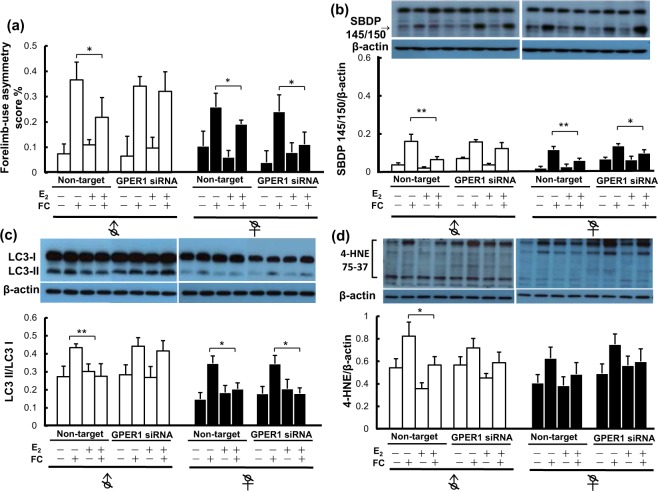
Silencing of GPER1 diminished the effects of E_2_ on FC-induced behavioral deficits, striatal injury, LC3 lipidation and lipid peroxidation in orchiectomized males but not in ovariectomized females. Castration was performed at 2 weeks before E_2_ implantation. E_2_ was implanted one day before iron infusion. GPER1 siRNA was injected twice into the striatum at 1 day before and 5 h after FC infusion. (**a**) Forelimb-use asymmetry score. (**b**) Level of SBDP 145/150. (**c**) LC3 lipidation. (**d**) Level of 4-HNE. Data are expressed as the means ± s.e.m. (n = 6). **Indicates *P* < 0.01; *Indicates *P* < 0.05.

To elucidate whether there is a sexually dimorphic role of GPER1 in mediating the effects of E_2_ on FC-induced injury and autophagy, we used GPER1 siRNA to silence GPER1. The efficiency of GPER1 siRNA silencing was verified by Western blot analysis (as shown in Supplement S3a). The results showed that E_2_ decreased the levels of FC-induced behavior deficits (Fig. 4a), spectrin cleavage (Fig. 4b), LC3 lipidation (Fig. 4c), and lipid peroxidation (4-HNE) (Fig. 4d) in the striatums of castrated male and female mice treated with non-target siRNA. GPER1 siRNA significantly diminished the suppressive effects of E_2_ on FC-induced behavior deficits (Fig. 4a) and spectrin cleavage (Fig. 4b) in castrated males but not in castrated females. GPER1 siRNA also significantly diminished the suppression of FC-induced LC3 lipidation and lipid peroxidation conferred by E_2_ in castrated males but not in castrated females (Fig. 4c,d).

**Figure 5:**
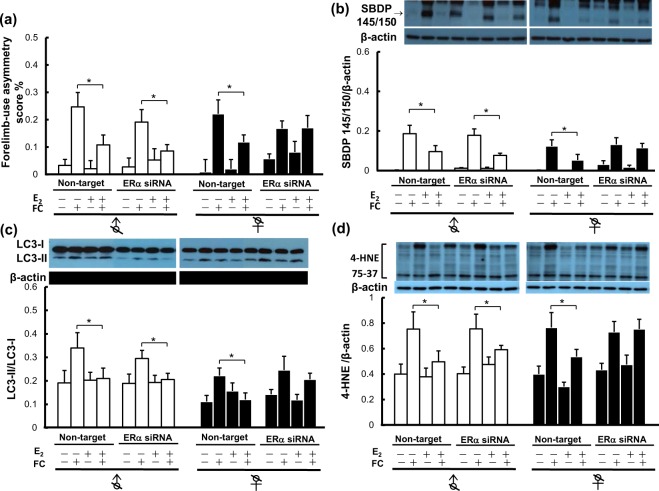
Silencing of ERα diminished the effects of E_2_ on FC-induced behavioral deficits, striatal injury, LC3 lipidation and lipid peroxidation in ovariectomized females but not in orchidectomized males. ERα siRNA was injected twice into the striatum at 1 day before and 5 h after FC infusion. (**a**) Forelimb-use asymmetry score. (**b**) Level of SBDP 145/150. (**c**) LC3 lipidation. (**d**) Level of 4-HNE. Data are expressed as the means ± s.e.m. (n = 6). **Indicates *P* < 0.01; *Indicates *P* < 0.05.

Additionally, to further understand whether ERα has a sexually dimorphic mediating role in the mechanism underlying neuroprotection by E_2_ against iron toxicity, we used ERα siRNA to silence ERα, and the knockdown efficiency of ERα siRNA was checked by Western blot analysis (as shown in Supplement S3b). The results showed that E_2_ decreased FC-induced behavior deficits (Fig. 5a), spectrin cleavage (Fig. 5b), LC3 lipidation (Fig. 5c), and lipid peroxidation (4-HNE) (Fig. 5d) in the striatums of castrated male and female mice treated with non-target siRNA. ERα siRNA significantly diminished the protective effect of E_2_ on FC-induced behavior deficits (Fig. 5a), spectrin cleavage (Fig. 5b), LC3 lipidation (Fig. 5c) and lipid peroxidation (Fig. 5d) in castrated females but not in castrated males.

## Discussion

The present study demonstrated that FC infusion triggers higher protein expression of GPER1 in the striatum of intact male mice than in females. A GPER1 agonist (G1) significantly decreased FC-induced LC3 lipidation only in castrated males. And GPER1 siRNA diminished the suppressive effect of E_2_ on FC-induced LC3 lipidation and lipid peroxidation in castrated males but not in castrated females. On the other hand, an ERα agonist (PPT) significantly decreased the level of FC-induced behavior deficits only in castrated females. While ERα siRNA diminished the suppressive effect of E_2_ on FC-induced behavior deficits, spectrin cleavage, LC3 lipidation and lipid peroxidation in a female-specific manner. These results suggest that GPER1 predominantly mediates the suppressive effect of E_2_ on FC-induced autophagy and injury in a male-specific manner, while ERα plays a dominant role in anti-oxidation and neuroprotection among females.

After ICH, men have higher mortality and worse survival than premenopausal women^1^. Although a sexually dimorphic therapeutic effect of tirilazad mesylate on the mortality of hemorrhagic patients was reported in 2007^11^, no effect on clinical outcome was observed^12^. The present management of ICH is mainly supportive, including maintenance of homeostasis and treatment of brain edema^13^. Estrogens contribute to the sex difference in ICH response^14^ due to their protective actions such as blocking lipid peroxidation reactions^15^; donating hydrogen atoms^16^; scavenging free radicals; attenuating NADPH oxidase activation; decreasing superoxide and reactive oxygen species generation; and reducing oxidative stress^17^. However, chronic administration of estrogens may elicit unwanted side effects including increased risk of breast/endometrial cancer, and feminization in males. Present results show that the protein level of ERα was significantly higher in intact female mice than in males and ERα siRNA diminished the effects of E_2_ on FC-induced lipid peroxidation in females but not in males. It suggests that endogenous estrogen in females may protect the striatum from iron overload predominantly by diminishing oxidative stress via ERα. The induction of GPER1 or ERα by FC-infusion is higher in males than in females, suggesting that a greater endogenous counter reaction against iron toxicity through estrogen receptors is required in males than in females. It may be explained by the fact that neural tissue was more sensitive to iron-induced brain injury in male than in female^18^. In males, the upregulation of GPER1 in the striatum implies a dominant stress-responsive role of GPER1 exists in males, who lack the endogenous protection by estrogen signaling via ERα. Furthermore, only in castrated males, G1 simulates the suppressive effect of E_2_ on FC-induced autophagy, while GPER1 siRNA diminishes the suppressive effect of E_2_ in FC-induced autophagy. These results suggest that GPER1 mediates the suppressive effect of E_2_ on the FC-induced autophagy that plays a male-specific harmful role in striatal injury. Although the effect of E_2_ on FC-induced lipid peroxidation in males may not be excluded, the present results show the sexually dimorphic mediating roles of GPER1 and ERα in the protective effect of E_2_ against FC-induced striatal injury. Our findings open the prospect for a male-specific autophagy suppression targeting the downstream signaling molecule, GPER1, of E_2_ for striatal injury caused by iron overload without the risk of feminization.

Iron accumulation plays a critical role in ICH-induced brain injury based on the findings that an iron chelating agent, such as deferoxamine, reduces ICH-induced brain edema, neuronal death, brain atrophy, and neurological deficits^19–21^. Several hours after ICH, edema forms after clot retraction. Subsequent lysis of red blood cell results in cleavage of hemoglobin by heme oxygenase-1 (HO-1) to carbon oxide and free toxic ferrous iron^22^. The toxicity of ferrous iron is based on Fenton chemistry where iron reacts with reactive oxygen intermediates the production of highly reactive free radical species. Reduction of the oxidized form of iron regenerates the Fenton active form of iron which re-enter the redox cycling. Thus, a continuous production of free radicals such as hydroxyl radicals is highly neurotoxic^23^. Animal studies show that intracerebral infusion of ferrous iron induces lipid peroxidation^24^ and increases the number of degenerated neurons^23^. It implies that accumulation of ferrous iron causes oxidative damage to brain cells and contributes to the long-term neurological deficit after hemorrhage. Moreover, Infusion of ferrous iron into rat striatum resulted in autophagy, and deferoxamine significantly reduced the ICH-induced autophagy suggesting a significant role of iron in ICH-induced autophagic cell death^25,26^. Our previous result also showed that FC induced a higher levels of autophagy^27^ and autophagic cell death in male than in female mice^5,6^. Mouse model of FC-infusion fully simulates the sex dimorphism in both injury severity and autophagy after ICH, and its injury severity is more homogeneous than the model of autologous blood infusion. The present study used the mouse model of FC-infusion instead of model of autologous blood infusion to address the male-specific GPER1 mediation in autophagy suppression by E_2_. We found that GPER1 and ERα play sexually dimorphic roles in protecting striatum against FC-induced brain injury.

E_2_ has been suggested to be a potential therapeutic agent for ICH^14,28^ and iron-induced brain edema *in vivo* and neuronal death *in vitro*^29^. A recent report indicated that ERα-mediated rapid estrogen signaling is involved in the neuroprotection activity of estrogen against oxidative toxicity^30^. GPER1 also mediates the neuroprotective effects of estrogen both *in vitro*^31^ and *in vivo*^32^. However, the molecular mechanism underlying the mediating role of GPER1 in autophagy suppression by E_2_ remains unknown. Previous studies have indicated that the GPER1 agonist G1 simulates the neuroprotective effect of E_2_ via the mTOR pathway^33^. G1 also protects cognitive function via activation of class I phosphoinositide 3-kinase (PI3K)/Akt-signaling^32^. The PI3K/Akt pathway keeps mTORC1 (a component complex of the mTOR pathway that inhibits autophagy) active^34^. High mTOR activity prevents activation of Ulk1 (a serine/threonine protein kinase that is essential for the initial stage of autophagy) by phosphorylating Ulk1 Ser 757 and disrupting the interaction between Ulk1 and AMPK, which directly activates Ulk1, through phosphorylation of Ser 317 and Ser 777^35^. In addition to directly inhibiting autophagy by disrupting the Ulk1-AMPK interaction, mTOR inhibits nuclear translocation of transcription factor EB (TFEB), a member of the bHLH leucine-zipper family of transcription factors that drives expression of autophagy-related genes (*atg4*, *atg9*, *bcl2*, *lc3*, *p62*, *uvrag* and *wipi*)^36^. TFEB works in conjunction with ZKSCAN3 (a master transcriptional repressor of autophagy), which represses the transcription of several autophagy-related genes (*lc3*, *ulk1*, *wipi2*, *atg9*)^37^. Accordingly, both PI3K/Akt/mTOR signaling and mTOR-dependent transcriptional regulation of autophagy-related genes may be involved in the mechanism underlying autophagy suppression by E_2_ via GPER1.

In summary, the present study has shown that GPER1 mediates the suppressive effects of E_2_ on FC-induced autophagy and injury in males, while ERα mediates the suppressive effects of E_2_ on FC-induced lipid peroxidation and injury in females. Activation of GPER1 plays a beneficial role for suppressing FC-induced autophagy only in males. This finding opens the prospect for a male-specific autophagy suppression targeting GPER1 activation for patients suffering from striatum iron overload caused by ICH.

## Materials and Methods

### Animals

A total of 384 C57BL/6 mice (192 males and 192 females) purchased from National Laboratory Animal Center, Taipei, Taiwan, was used and no animal was excluded. To study the sex differences in constitutive and inducible estrogen receptors, we used intact C57BL/6 mice at 12 weeks of age. To study the contribution of exogenous E_2_ to the sex difference in FC-induced striatal injury, we castrated both male and female C57BL/6 mice for two weeks to exclude the influence from endogenous sex hormones, and then implanted them with estradiol (E_2_). According to our previous study, implantation of an E_2_ Silastic tube released E_2_ in serum at physiological levels (56–92 pg/ml) and were maintained for at least 7 days^38^. A Silastic tube (2 mm outer diameter, 1 mm inner diameter; 20 mm in length) containing 0.8 mmol of E_2_ (Sigma-Aldrich, E8515) was implanted subcutaneously 24 h before FC infusion^6^. Alternatively, a Silastic tube filled with 1.6 mmol of GPER1 agonist G1 (Cayman; CAS Registry No. 881639-98-1) or with 1.6 mmol of ERα agonist PPT (propyl pyrazole triol; Cayman, CAS Registry No. 263717-53-9) was implanted subcutaneously at 24 h before FC infusion to simulate the specific activation of GPER1 or ERα, respectively. Three microliters of fresh prepared FC (1 nmol/μl) (Ammonium iron sulfate: Sigma, Cat. No. 21540-6; Citric acid: Amresco, Cat. No. 0101) was infused into the right striatum (coordinates: 0.2 mm anterior, 2.5 mm lateral, and 3.5 mm ventral to the bregma) using a microinfusion pump (CMA Microdialysis, Sweden) at a rate of 1 μl/min. All animals were randomly assigned to control or experiment groups. The iron deposition was confirmed by Prussian blue assay on brain sections from mice infused with FC as shown in Supplement S4. Mice of normal saline-infusion were used as the control group. Two days after FC infusion, the forelimb-use asymmetry test was performed before sacrifice. Then, the samples for Western blot were dissected at 2 mm anterior and 2 mm posterior of the injection site, then separated the outer cortex and isolated the striatum. All operations were performed under anesthesia with Zoletil 50 (1 ml/kg body weight intraperitoneally). All experiments were approved by the Kaohsiung Medical University Committee for the Use of Experimental Animals (IACUC approved No: 102117).

### Administration of siRNA

GPER1 siRNA (1000 nM) or ERα siRNA (1000 nM) was mixed with an equal volume of Invivofectamine 3.0 (Invitrogen, Cat. No. IVF3001) and was injected twice (5 μl each time) into the right striatum: once at 24 hours before and once at 5 hours after FC infusion. Two days later, tissue samples containing the striatum were sampled for the detection of silencing efficiency of GPER1 or ERα by Western blot analysis. The mouse GPER1 siRNA mixture contains three Stealth siRNA sequences (MSS233774, MSS233775, MSS233776) (Invitrogen, Cat. No. 1320001); Stealth RNAi negative control duplexes (Invitrogen, Cat. No. 12935-300) were used as a non-target siRNA for GPER1. The sequences of ERα siRNA were 5′-CUGGUUCAUAUGAUCAACUGG-3′ and 3′-AGUUGAUCAUAUGAACCAGCU-5′. Silencer negative control siRNA (Invitrogen, Ambion, Cat. No. AM4611) was used as a non-target siRNA for ERα.

### Forelimb-use asymmetry test

To assess behavior deficits due to striatal injury, we evaluated forelimb-use asymmetry scores two days after FC infusion. Each individual mouse was placed in a transparent cylinder (25 cm in diameter and 30 cm in height) in the dark, and the use of ipsilateral limbs (I), contralateral limbs (C), or simultaneous use of both forelimbs (B) was observed for a 5-minute period. The test was randomized, blind, and repeated twice in each mouse. The forelimb-use asymmetry score was calculated using the following equation:

$$[{\rm{I}}/({\rm{I}}+{\rm{C}}+{\rm{B}})]-[{\rm{C}}/({\rm{I}}+{\rm{C}}+{\rm{B}})].$$

### Western blot analysis

Tissue samples were homogenized in 5 volumes of lysis buffer containing 50 mmol/L Tris base, 150 mmol/L NaCl, 5 mmol/L EDTA, 50 mmol/L NaF, 0.1 mmol/L Na_3_VO_4_, 1% Triton X-100, pH 7.4, and protease inhibitor. Homogenates were clarified by centrifugation at 13000 rpm for 30 min, and the concentration of total soluble protein was measured by using a commercially available dye reagent (Protein Assay Kit II, Bio-Rad, Hercules) with bovine serum albumin as a standard. Protein samples were loaded on an 8 or 12% polyacrylamide gel for electrophoresis (SDS-PAGE; Bio-Rad, Alcobendas, Spain) and then transferred to a PVDF membrane. After being blocked with TBS-TWEEN 20 (0.05%) containing 5 or 10% nonfat milk for 1 h, the membranes were incubated overnight at 4 °C with the following primary antibodies: (i) rabbit polyclonal antibodies against LC3B (Sigma, Cat. No. L7543) at 1 mg/mL; (ii) mouse monoclonal antibody raised against amino acids 2368–2472 of human αII-spectrin (Santa Cruz, Cat. No. sc-48382) at 1/1000 dilution; (iii) rabbit polyclonal antibodies against GPER1 (Abcam, Cat. No. ab39742) at 1/250 dilution; (iv) rabbit monoclonal anti-ERα antibody, clone 60 C (Millipore, Cat. No. 04–820); and (v) primary antibody against 4-HNE (Abcam, Cat. No. ab48506). Then, the membranes were incubated in TBS-TWEEN 20 (0.05%) containing 10% nonfat milk for 1 h. The primary antibodies were revealed with horseradish-peroxidase-labeled goat anti-rabbit IgG secondary antibodies (Thermo, Cat. No. 31460) or peroxidase-conjugated AffiniPure goat anti-mouse IgG secondary antibodies (Jackson, Cat. No. 115-035-003) and were detected with enhanced chemiluminescence reagent. Each blots showed in figures are derived from the same gel with margin-cropping.

### Statistics

FC-induced injury and E_2_ neuroprotection were compared between brains from males and females using a two-way ANOVA followed by a *post hoc* Scheffe test. Data on the effects of E_2_, G1 or PPT, GPER1 siRNA, and ERα siRNA on FC-cytotoxicity, autophagy or lipid peroxidation were analyzed using a multiway ANOVA to determine the effect of each factor. Significance was accepted at p < 0.05.

## Supplementary information

Targeting GPER1 to suppress autophagy as a male-specific therapeutic strategy for iron-induced striatal injury
