# Supplementary material for: Targeting GPER1 to suppress autophagy as a male-specific therapeutic strategy for iron-induced striatal injury
Source: Sci Rep. 2019 Apr 30;9:6661. doi: 10.1038/s41598-019-43244-0 (PMC6491488; doi:10.1038/s41598-019-43244-0)
Supplement: Supplementary file 1 — Targeting GPER1 to suppress autophagy as a male-specific therapeutic strategy for iron-induced striatal injury [file 41598_2019_43244_MOESM1_ESM.docx]

**Targeting GPER1 to suppress autophagy as a male-specific therapeutic strategy for iron-induced striatal injury**

Tzu-Yun Chen, MS^1^; Chih-Lung Lin, MD, PhD^2^; Li-Fang Wang, PhD^3^; Ke-Li Tsai, PhD^1^; Jun-Yu Lin, MS ^1^, Chin Hsu, PhD* ^1, 4, 5^

**Supplement S1**

**No effect of castration on protein levels of GPER1 and ERα in both males and females. (a) GPER1; (b)** **ERα.** Orchiectomy or ovariectomy was performed at 2 weeks before the sampling of brain tissue. Data are expressed as the means ± s.e.m. (n=12).

**Supplement S2**

**Serum levels of E_2_ in castrated male and female mice with or without E_2_ implantation.** Castration was performed at 2 weeks before E_2_ implantation. Silastic tubes containing E_2_ were implanted one day before FC infusion. Two days after FC infusion, serum was sampled and the level of E_2_ was measured by using an ELISA kit. Data are expressed as the means ± s.e.m. (n=6). ** indicates *P*< 0.01.

**Supplement S3**

**Silencing effects of GPER1 siRNA and ERα siRNA on protein levels of GPER1 (a) and ERα (b), respectively.** Two 5 μl doses of siRNA (500 nM) or ERα siRNA (500 nM) were administered: one at 24 h before and one 6 h after FC infusion. Data are expressed as the means ± s.e.m. (n=6).

**Supplement S4**

**The Prussian blue assay on brain section from mice infused with or without ferrous citrate (FC).** Two days after FC infusion, the brain tissue containing striatum was sectioned and stained by working solution which is a mixture of equal parts of hydrochloric acid and potassium ferrocyanide prepared immediately before use. Arrows in the lower left panel indicate dark blue deposits that confirmed the iron deposition after FC infusion.
